# Impurity Shielding in Li/Al-LDH-Based Lithium Recovery from Produced Water via Activated Carbon Pretreatment

**DOI:** 10.1021/acssuschemeng.5c05254

**Published:** 2025-08-25

**Authors:** Yanan Pan, Wencai Zhang

**Affiliations:** Department of Mining and Minerals Engineering, 1757 Virginia Polytechnic Institute and State University, Blacksburg, Virginia 24061, United States

**Keywords:** produced water, Li/Al-LDH, activated carbon pretreatment, structural stabilization, lithium selectivity, green extraction

## Abstract

Produced water (PW), a complex industrial wastewater byproduct, contains diverse organic contaminants and competing ions that hinder lithium recovery via lithium/aluminum-layered double hydroxide (Li/Al-LDH) adsorption. In this study, an activated carbon (AC)-based pretreatment strategy was developed to improve Li^+^ adsorption efficiency by mitigating impurity-induced interference. Two commercial ACs were screened across three PW sources, with optimal dosage and contact time determined for each. AC pretreatment effectively removed key organic compounds and divalent cations, enhancing the aqueous environment for lithium uptake. More importantly, it preserved the structural integrity of Li/Al-LDH, reducing degradation associated with coadsorbed organics. Adsorption experiments confirmed increased Li^+^ capacity following AC treatment, while adsorption kinetics remained unchanged. Li^+^ selectivity was also modestly improved, particularly over Ca^2+^ and Mg^2+^. Rather than directly enhancing Li^+^ adsorption, AC served as a protective pretreatment, indirectly enabling more efficient and stable lithium recovery. This impurity-shielding approach offers a scalable and environmentally benign pathway for critical mineral recovery from PW, aligning with sustainable water treatment and circular economy principles.

## Graphical abstract

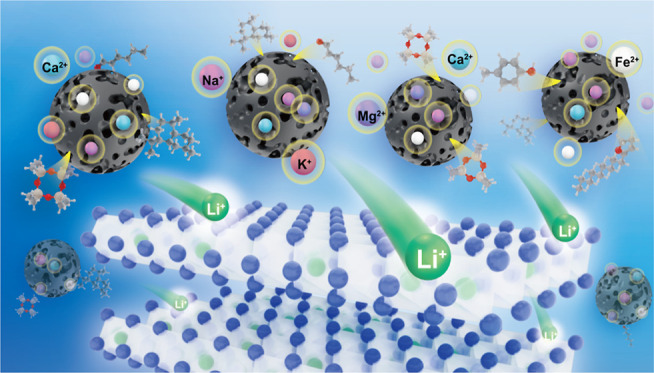

## Introduction

With growing concerns over environmental sustainability and responsible water management, industries are increasingly focusing on minimizing waste and optimizing resource utilization. Produced water (PW) is an unavoidable byproduct of oil and gas production, consisting of water that naturally exists alongside hydrocarbons in underground reservoirs and is brought to the surface during extraction.^1^ Its composition varies depending on geological formation but generally contains mineral salts, organic compounds, heavy metals, naturally occurring radioactive materials, and large amounts of critical minerals.^2,3^ With global production estimated at around 250 million barrels per day, PW represents the largest waste stream in the oil and gas sector.^4^ Due to its vast volume and the presence of valuable dissolved minerals, PW is increasingly viewed as a potential resource rather than a waste product. In recent years, the U.S. Department of Energy (DOE) has recognized its significance for critical mineral recovery, promoting research into more sustainable extraction and resource utilization methods.^5^

Lithium, a lightweight alkali metal, is valued for its high electrochemical potential and energy density, making it an essential element in rechargeable lithium-ion batteries that power consumer electronics, electric vehicles, and grid-scale energy storage systems.^6,7^ Recognizing its irreplaceable role in battery technology, the U.S. Geological Survey (USGS) has classified lithium as a critical mineral.^8^ Traditionally, lithium extraction relies on Salt Lake brines and hard rock mining from minerals such as spodumene, petalite, and lepidolite.^9,10^ However, these conventional sources are insufficient to meet the rapidly growing global demand, with supply shortages anticipated as early as 2030.^11^ To address this challenge, alternative lithium sources are crucial for diversifying supply chains and ensuring long-term resource availability. PW often contains dissolved lithium, offering a valuable yet underutilized resource. Unlike conventional lithium sources, lithium extraction from PW presents a dual advantage: it not only helps mitigate the environmental and disposal challenges associated with PW but also contributes to strengthening lithium supply security.^12^

Multiple technologies exist for extracting lithium from PW, such as solvent extraction,^13^ membrane separation,^14^ adsorption,^15^ electrochemical recovery,^16^ and precipitation/ion-exchange.^17^ While these approaches offer potential, many face challenges in scaling up. For instance, membrane separation is prone to fouling, electrochemical recovery demands substantial energy input, and solvent extraction involves environmentally hazardous organic solvents. Adsorption technology has emerged as a promising alternative due to its distinct advantages. It demonstrates exceptional selectivity for Li^+^ ions, even in the presence of competing ions.^18^ Additionally, adsorption requires minimal energy compared to other methods and avoids the ecological risks linked to solvents. The reusability of adsorbents further bolsters its appeal, as regenerating these materials reduces both operational costs and waste generation. These attributes position adsorption as a scalable and sustainable option for lithium recovery from PW, aligning with industrial needs for cost-effectiveness and environmental responsibility. Lithium/aluminum layered double hydroxide (Li/Al-LDH) is one of the most widely studied lithium adsorbents, distinguished by its environmental advantages, including easy synthesis and lithium desorption under neutral conditions.^19,20^ The chemical formula of Li/Al-LDH is represented as mLiCl·2Al­(OH)_3_·*n*H_2_O (0 < *m* < 1). Structurally, Al–O octahedra share edges to form a two-dimensional layered framework, where Al atoms occupy two-thirds of the octahedral sites, leaving the remaining one-third as accessible cavities for the selective insertion and extraction of Li^+^ ions (eq 1). This unique structure enables high lithium selectivity and efficient recovery, making Li/Al-LDH a promising material for sustainable lithium extraction.

1 $$xLiCl+\left(m-x\right)LiCl\cdot 2Al{\left(OH\right)}_{3}\cdot \left(n+1\right)H_{2}O↔mLiCl\cdot 2{Al\left(OH\right)}_{3}\cdot nH_{2}O+H_{2}O$$

In our previous studies, optimized Li/Al-LDH was synthesized and applied for lithium extraction from PW sourced from the Marcellus Formation. This adsorbent demonstrated good reusability, maintaining a stable lithium adsorption capacity of approximately 2.5 mg/g over five cycles.^21^ Additionally, various modification strategies have been explored to enhance the lithium extraction performance of Li/Al-LDH in PW, such as poly­(acrylic acid) and polyethylene glycol functionalization.^22,23^ However, due to the low initial lithium concentration and the complex chemical composition of PW, the layered structure of Li/Al-LDH is susceptible to degradation by organic matter and competing ions, which can reduce lithium recovery efficiency and shorten the adsorbent’s operational lifespan. Therefore, developing an efficient, easy-to-implement, and environmentally friendly approach to mitigate the adverse effects of impurities in PW on the Li/Al-LDH lithium adsorption process remains a critical focus.

Activated carbon (AC) is widely recognized for its exceptional adsorption capacity, large surface area, and well-developed porous structure, making it highly effective in removing organic contaminants and competing ions from wastewater.^24^ Building on our prior work with Li/Al-LDH, this study develops a dual-function strategy combining AC pretreatment with Li/Al-LDH adsorption for lithium recovery from PW—a highly challenging source with low lithium concentration, high salinity, and organic interference. We systematically optimized AC type, dosage, and treatment time, and evaluated its impact using UV–vis, GC–MS, and structural characterizations (XRD, FTIR, SEM). Results show that AC effectively removes fouling species and protects Li/Al-LDH from structural degradation. Kinetic and selectivity studies further confirm that lithium uptake is improved not by altering the adsorbent’s properties, but by reducing environmental interference. Compared to other sorbents, Li/Al-LDH offers low cost, scalability, and mild regeneration conditions, making the combined process industrially viable. This work advances a sustainable and practical approach for lithium extraction from unconventional resources.

## Methodology

### Materials

Raw materials for the synthesis of Li/Al-LDH, including LiCl (≥99 wt % purity), AlCl_3_·6H_2_O (>99 wt % purity), and NaOH (≥97 wt % purity), were all purchased from Thermo Fisher Scientific and directly used without any purification. Li/Al-LDH was synthesized through a one-step coprecipitation approach.^25^ A mixed aqueous solution containing LiCl and AlCl_3_·6H_2_O was gradually introduced into a NaOH solution under continuous stirring. The reaction progression was monitored through pH measurements, with termination occurring upon reaching a predetermined end point. The resulting precipitate underwent multiple washing cycles with deionized water (DIW), followed by drying at 60 °C to obtain the final Li/Al-LDH product.

Coconut-shell activated carbon (CAC) and bituminous-coal activated carbon (BAC), both in 12 × 40 mesh granular form, were obtained from Fresh Water System and used without further pretreatment. These two types of activated carbon were selected due to their distinct physicochemical characteristics and broad representation of biomass-derived (CAC) and fossil-based (BAC) origins. CAC typically features high microporosity and low ash content, making it effective for organic matter removal, while BAC provides a wider pore size distribution, facilitating the removal of both organics and competing ions. Their widespread use in industrial water treatment also supports the practical relevance of this study. PW samples were collected from three distinct sources within the Marcellus Shale formation, USA: (1) a 50,000-gallon storage tank at a water treatment facility; (2) a production well with 13–14 years of operational history; and (3) a relatively new well that had been in service for 1–2 years. Key water quality parameters, including total organic carbon (TOC), pH, total dissolved solids (TDS), and Li^+^ concentration, are summarized in Table 1. Additional ionic composition data are provided in Table S1 of the Supporting Information.

**Table 1:** TOC, pH, TDS, and Li^+^ Concentration of Three PW Samples^a^

	TOC (mg/L)	pH	TDS (ppt)	Li^+^ (mg/L)
Tank-produced water (TPW)	47.54	5.84	105.7	50
Old well-produced water (OWPW)	20.22	5.33	176.6	81
New well-produced water (NWPW)	18.81	5.71	174.9	80

a The abbreviation “ppt” refers to parts per thousand.

To further characterize the organic composition of the PW samples, Table S2 presents a qualitative comparison of the primary organic constituents in each sample, inferred from their field source and operational age. These compositional differences served as the basis for selecting appropriate AC types and dosages to reduce organic interference and improve lithium recovery efficiency.

### Pretreatment Experiments

A systematic evaluation of both CAC and BAC was conducted in PW pretreatment experiments. The study aimed to determine three critical parameters: the optimal AC type, the precise AC dosage, and the effective AC contact duration. To achieve this, varying AC dosages were tested at 0 g/100 mL, 0.5 g/100 mL, 1 g/100 mL, 2 g/100 mL, 4 g/100 mL, and 6 g/100 mL. Each AC and PW mixture was continuously stirred and reacted to facilitate the removal of organic matter. The experiment was repeated using different AC types and PW samples to identify the most effective AC type and dosage for specific PW conditions. The effectiveness of pretreatment was subsequently evaluated by comparing lithium adsorption capacity using Li/Al-LDH adsorbents in parallel systems-pretreated versus untreated PW. Additionally, TOC measurements were conducted before and after AC treatment at different dosages to calculate the Organic Matter Removal Efficiency (OMRE, %). The formula used for OMRE calculation is provided below

2 $$OMRE=100\%\times \frac{{TOC}_{initial}-{TOC}_{final}}{{TOC}_{initial}}$$

where TOC_initial_ (mg/L) means the TOC value in the untreated PW samples, while TOC_final_ (mg/L) refers to the TOC value in the treated PW samples. AC also exhibits a certain binding capacity for metal ions in PW, which can be defined as the Ion Removal Efficiency (IRE, %)

3 $$IRE=100\%\times \frac{C_{before}-C_{after}}{C_{before}}$$

where *C*_before_ (mg/L) means the ion concentration in the untreated PW samples, while *C*_after_ (mg/L) refers to the ion concentration in the AC-treated PW samples.

After determining the optimal AC type and dosage, various contact durations were tested at 0, 10, 30, 50, 90, and 120 min to identify the most effective treatment time. The effectiveness of the pretreatment process was evaluated by comparing the lithium adsorption capacity of Li/Al-LDH adsorbents in treated versus untreated PW samples. During the process, the LDH solids to PW liquid ratio is 1 g/100 mL, with the duration fixed at 2 h. The lithium adsorption capacity (*q*, mg/g) can be calculated by testing the Li^+^ concentration in the PW samples before and after LDH adsorption, using eq 4.

4 $$q=\frac{v\times \left(C_{0}-C_{f}\right)}{m}$$

where *C*_0_ (mg/L) and *C*_f_ (mg/L) refer to the Li^+^ ion concentration in the PW samples before and after the LDH adsorption, respectively. *v* (L) is the volume of the feed PW samples and *m* (g) is the added mass of LDH powder.

### Lithium Adsorption

During the lithium adsorption process using Li/Al-LDH in PW, a certain amount of organic matter may also be adsorbed into the Li/Al-LDH structure, leading to structural degradation. This phenomenon is defined as Organic Uptake Index (OUI, %). The calculation formula is as follows

5 $$OUI=100\%\times \frac{{TOC}_{initial,Li}-{TOC}_{final,Li}}{{TOC}_{initial,Li}}$$

where TOC_initial,Li_ (mg/L) and TOC_final,Li_ (mg/L) refer to the TOC values in PW before and after LDH adsorption. Although not a direct measure of structural degradation, the OUI can serve as a proxy indicator for the potential organic-induced damage to LDH.

To evaluate the impact of AC pretreatment on lithium adsorption performance across different PW systems, adsorption kinetics and selectivity studies were conducted. Adsorption kinetics experiments were performed with a fixed Li/Al-LDH powder-to-PW ratio of 1*g*/100 mL. All experiments were carried out in a bath shaker at a constant shaking speed of 150 rpm. Lithium adsorption capacity was determined at various time intervals (0, 2, 5, 10, 25, 40, 60, 90, and 120 min) using eq 4. The experimental data were then analyzed by fitting them to both the pseudo-first-order and pseudo-second-order kinetic models, as described by eqs S1 and S2 respectively. For lithium adsorption selectivity in these PW samples, the distribution coefficient *K*_Me_ (mL/g) and selectivity factor *a*_Li_^Me^ were calculated as eqs S3 and S4.

### Assays

The surface area and pore structure of BAC and CAC were characterized using N_2_ adsorption–desorption isotherms at 77 K, measured with a Micromeritics 3Flex analyzer. The Brunauer–Emmett–Teller (BET) method was employed to determine the specific surface area, while the Barrett–Joyner–Halenda (BJH) method was used to analyze the pore size distribution.

TOC concentrations of PW samples following different treatments were quantified using a SHIMADZU TOC-L analyzer (Shimadzu Corporation, Japan). Ion concentrations in solutions were measured by ICP–MS (Agilent 8900, Agilent Technologies, USA). Besides, the organic matter content in PW was analyzed using a UV–vis spectrophotometer (Agilent, USA). To further elucidate the composition of organic compounds, GC–MS was performed using an Agilent 7890 GC/5975 MS system equipped with an HP-5 MS capillary column (30 m × 0.25 mm × 0.25 μm). High-purity helium was used as carrier gas at a flow rate of 1 mL/min, with a split ratio of 50:1. The initial oven temperature was maintained at 28 °C for 3 min, then ramped to 180 °C at 5 °C/min. Organic compounds were identified using the NIST 08 library.

The crystalline structure of Li/Al-LDH was investigated using XRD over a 2θ range of 5° to 80°, with Ni-filtered Cu Kα radiation. FTIR spectroscopy was conducted using a Thermo Fisher Scientific Nicolet instrument, with samples prepared as KBr pellets. SEM was utilized to examine the morphology of the LDH samples.

## Results and Discussion

### Activated Carbon Screening and Pretreatment Optimization

As both ACs used in this study are commercial products, comprehensive characterization of their surface and porous properties was conducted, including analyses of specific surface area, pore volume, and pore size distribution. As given in Figure S1, BAC predominantly features microporous structures, whereas CAC contains more extensive mesoporous networks. Both materials maintain well-developed porosity suitable for organic matters removal applications. The comparative efficiency of BAC and CAC in organic matter removal was then systematically assessed across varying AC dosages in the TPW system (Figure 1a). At a lower AC dosage (0.5 g/100 mL), BAC exhibits a clear advantage, achieving 30% organic matter removal efficiency, which is approximately 24% higher than that of CAC. However, this trend reverses with increasing carbon dosage, as CAC demonstrates a consistent improvement in removal efficiency that correlates directly with dosage increments. At the highest solid-to-liquid ratio (6 g/100 mL), CAC reaches its maximum removal efficiency of 62%, more than twice the plateaued performance of BAC (30%). This shift suggests that while BAC is more effective at lower dosages, CAC’s removal capacity scales more favorably with increased dosage, ultimately surpassing BAC in high-dosage applications. In the OWPW system (Figure 1b), BAC reaches its peak removal efficiency of ∼42% at 2 g/100 mL, outperforming CAC at this dosage. However, at lower solid-to-liquid ratios, CAC exhibits higher removal efficiency, indicating its effectiveness in lower-dosage applications. At higher AC dosages, the removal efficiencies of BAC and CAC become comparable, suggesting diminishing returns beyond a certain threshold. Considering both removal efficiency and material consumption, 2 g BAC/100 mL stands out as the optimal dosage, offering an effective balance between performance and material consumption. In the NWPW system (Figure 1c), CAC exhibits a more stable OMRE trend within the 1–4 g/100 mL range, reaching a peak removal efficiency of 48%. In contrast, BAC shows localized improvements at 4 g/100 mL and 6 g/100 mL, but its overall trend fluctuates more, indicating less consistent performance. Additionally, at higher AC dosages, material consumption increases significantly, making 1–4 g CAC/100 mL the preferred operational range for effective organic removal. However, the ultimate selection of the optimal AC type and dosage for different PW systems should also take into account the impact of AC pretreatment on subsequent lithium adsorption efficiency using Li/Al-LDH, ensuring optimum lithium recovery under varying AC pretreatment conditions.

**Figure 1:**
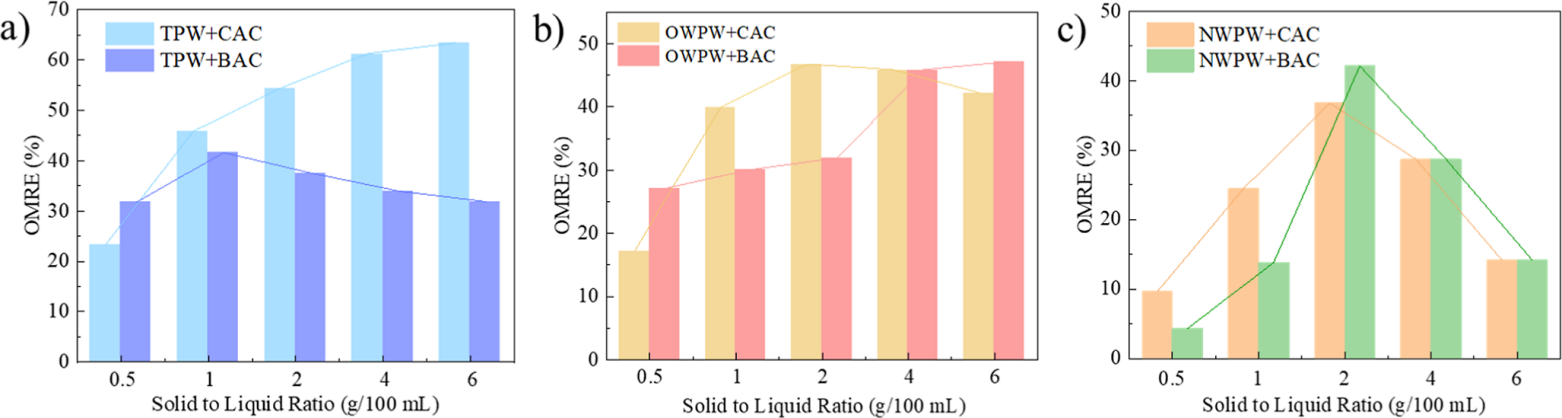
OMRE of BAC and CAC in the (a) TPW treatment, (b) OWPW treatment, and (c) NWPW treatment.

To determine the optimal AC type, dosage, and contact time, lithium adsorption capacity was used as a key evaluation metric in conjunction with the efficiency of organic matter removal from the different PW sources. Figure 2a presents the evaluation of lithium adsorption capacity of different ACs in TPW under a range of solid-to-liquid ratios. The results indicate that BAC pretreatment had minimal impact on enhancing lithium adsorption, whereas CAC pretreatment led to a significant increase in lithium adsorption with rising solid-to-liquid ratios, reaching optimal performance within the 2–6 g/100 mL range. Considering both adsorption efficiency and material consumption, 2 g CAC/100 mL was identified as the optimal dosage. Figure 2b presents the lithium adsorption performance in OWPW under different AC pretreatment conditions. Both BAC and CAC improved lithium adsorption, but BAC exhibited superior performance, with 2 g BAC/100 mL yielding the highest lithium adsorption. This can be attributed to the prolonged subsurface exposure of OWPW, which results in a higher concentration of humic substances, aromatic hydrocarbons, and other persistent organic compounds. The relatively broader pore size distribution of BAC, derived from bituminous coal, may facilitate the adsorption of these larger and more structurally complex organics. By adsorbing such compounds, BAC potentially reduces their competitive interaction with Li/Al-LDH, thereby improving lithium adsorption efficiency. In addition, Figure 2c illustrates lithium adsorption in NWPW under different AC pretreatment conditions, showing that 1 g CAC/100 mL was the most effective. Unlike OWPW, NWPW primarily contains short-chain hydrocarbons and crude oil residues, with relatively low dissolved organic matter (DOM) content due to its shorter exposure time in the formation. Given its mesoporous structure, CAC is likely more effective in removing short-chain hydrocarbons and surfactants, optimizing the aqueous phase environment and enhancing lithium adsorption by Li/Al-LDH. Unlike OWPW, NWPW does not require BAC’s microporous structure for the removal of complex humic substances and aromatic hydrocarbons, making CAC the more suitable choice. Based on the combined analysis of organic matter removal efficiency and lithium adsorption performance, the optimal AC selection and dosage for each PW system are determined as follows: 2 g CAC/100 mL for TPW, ensuring the effective removal of soluble organics and enhanced lithium adsorption; 2 g BAC/100 mL for OWPW, facilitating the removal of humic substances and aromatic hydrocarbons to reduce lithium adsorption interference; and 1 g CAC/100 mL for NWPW to efficiently remove short-chain hydrocarbons and surfactants, optimizing aqueous phase conditions for lithium adsorption. Importantly, it should be noted that lithium adsorption is not solely governed by organic matter removal efficiency, as it is also influenced by factors such as ionic strength, competing cations, and surface charge conditions of the Li/Al-LDH material.

**Figure 2:**
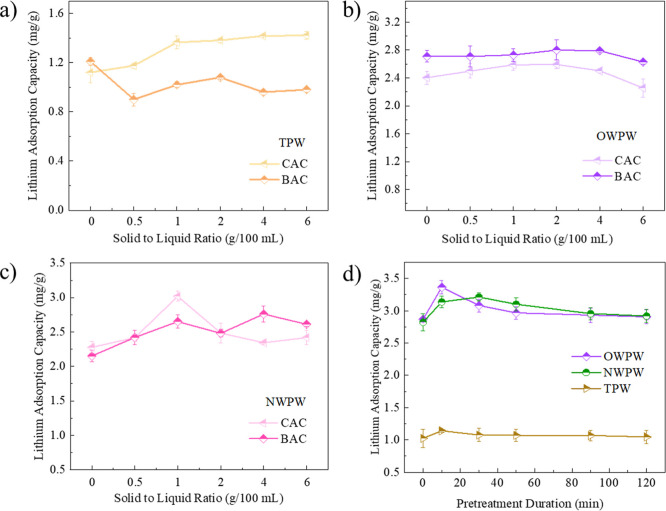
Lithium adsorption capacity of the different ACs at varying solid-to-liquid ratios in (a) TPW, (b) OWPW, and (c) NWPW; (d) Lithium adsorption capacity of the different PW samples over different pretreatment durations.

After determining the optimal AC type and dosage, the pretreatment duration for each PW system was further optimized under these specific conditions, as shown in Figure 2d. It is evident that TPW exhibits significantly lower lithium adsorption capacity, primarily due to its lower initial lithium concentration and higher organic pollutant content. In contrast, OWPW and NWPW achieve lithium adsorption capacities of approximately 3 mg/g, demonstrating their suitability for lithium extraction. Additionally, lithium adsorption across all three PW systems follows a trend of initial increase, followed by a decline, and eventual stabilization as pretreatment time extends. The highest lithium adsorption is observed at 10 min for TPW, 10 min for OWPW, and 30 min for NWPW, beyond which no further improvement is observed. This suggests that prolonging AC contact time does not enhance lithium adsorption efficiency. The primary reason is that, as the exposure time increases, the available adsorption sites on the activated carbon become progressively saturated, thereby limiting the further removal of organic matter. Once adsorption equilibrium is reached, extending the pretreatment duration yields minimal additional benefit for enhancing lithium adsorption. In conclusion, Table 2 summarizes the optimal AC type, dosage, and contact duration for the three PW samples. These optimized conditions will be applied in all subsequent experiments.

**Table 2:** Optimal AC Conditions for the Three PW Samples

	AC type	AC dosage	AC contacting duration
TPW	CAC	2 g/100 mL	10 min
OWPW	BAC	2 g/100 mL	10 min
NWPW	CAC	1 g/100 mL	30 min

### Comparative Analysis of Produced Water before and after AC Treatment

To better understand the changes in the properties of the three types of PW before and after treatment with different ACs, the samples were analyzed using UV–vis and GC–MS. As shown in Figure 3a, a distinct absorption peak near 290 nm is attributed to the n → π* electronic transition of the carbonyl group (–C=O).^26,27^ Furthermore, the absorbance of all three systems decreased following AC treatment, demonstrating the effectiveness of AC in removing organic pollutants. Among them, the CAC treatment in the NWPW system exhibited the most significant removal effect, as evidenced by the largest reduction in absorbance, indicating its superior capability in eliminating organic contaminants.

**Figure 3:**
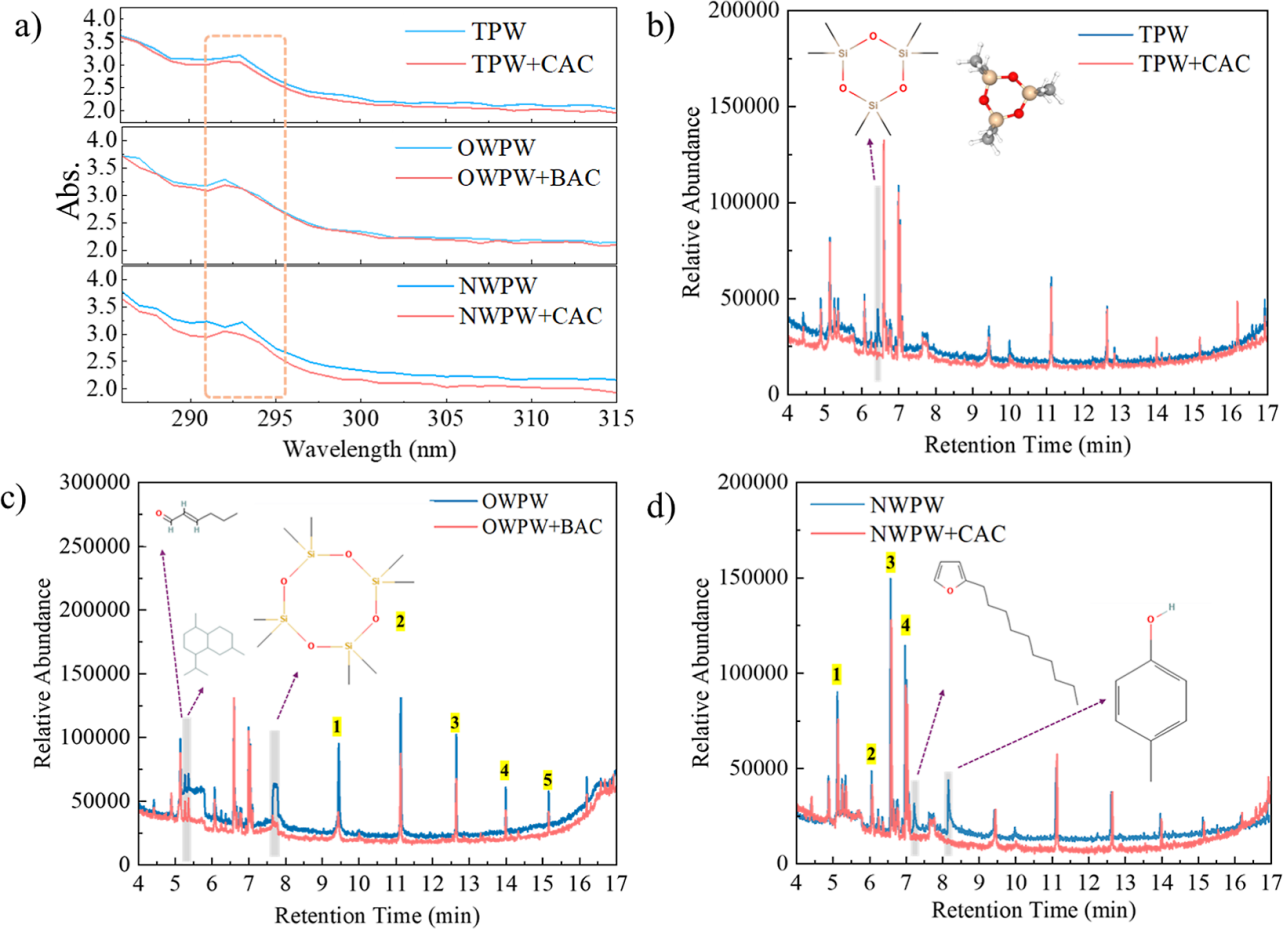
(a) UV–vis spectrum of different PW before and after AC treatment; (b) GC–MS spectrum of TPW and TPW after CAC treatment, (c) OWPW and OWPW after BAC treatment, and (d) NWPW and NWPW after CAC treatment.

Figure 3b–d presents the GC–MS spectra of the different PW systems before and after treatment with the most suitable AC as listed in Table 2. As shown in Figure 3b, after CAC treatment of TPW, the relative abundance of all detected peaks decreased, indicating a reduction in the concentration of most organic compounds. However, a new peak corresponding to C_6_H_18_O_3_Si_3_ appeared after CAC treatment. This may be due to changes in the chemical composition or matrix of the solution caused by CAC treatment, which potentially released or transformed previously undetectable trace siloxane compounds into detectable forms.^28^ In Figure 3c, following BAC treatment of OWPW, the relative abundance of compounds with retention times at 5.2 min (C_6_H_10_O), 5.3 min (C_15_H_28_), and 7.7 min (C_8_H_24_O_4_Si_4_) significantly decreased or were no longer detected. The removal of C_6_H_10_O, a six-carbon oxygen-containing compound such as cyclohexanone, is likely due to the strong adsorption capacity of BAC for oxygenated compounds, facilitated by its well-developed microporous and mesoporous structure.^29^ Similarly, the reduction or disappearance of C_15_H_28_, a long-chain alkane or terpenoid, can be attributed to van der Waals interactions, which promote the adsorption of hydrophobic hydrocarbons on BAC’s nonpolar surface.^30^ The removal of C_8_H_24_O_4_Si_4_, a siloxane compound, is likely driven by hydrophobic–hydrophobic interactions with BAC. Additionally, five other compounds, C_10_H_30_O_5_Si_5_, C_12_H_36_O_6_Si_6_, C_14_H_42_O_7_Si_7_, C_24_H_72_O_12_Si_12_, and C_28_H_57_NO_3_Si_2_, exhibited a noticeable reduction in concentration. These compounds primarily belong to the categories of organosilicon compounds, which originate from drilling fluids or degradation products of oilfield chemicals, and amino-functionalized siloxanes, commonly derived from oilfield additives such as surfactants or stabilizers. The observed reduction suggests that BAC may have facilitated their degradation or transformation through oxidation or cleavage into smaller organic molecules.^31,32^

Figure 3d presents the changes in organic compounds in NWPW before and after treatment with CAC. After CAC treatment, the compounds detected at retention times of 7.3 min (C_14_H_24_O) and 8.2 min (C_7_H_8_O) were no longer present. This is likely due to CAC’s well-developed porous structure, which enables the adsorption of C_14_H_24_O, an oxygen-containing compound, through polar interactions.^33^ Additionally, CAC’s strong π–π interactions contribute to the removal of aromatic compounds, explaining the elimination of C_7_H_8_O.^34^ In addition, the relative abundance of C_9_H_18_, C_9_H_16_O, C_10_H_2_O, and C_11_H_22_O_3_ decreased significantly after CAC treatment. These compounds may originate from crude oil components introduced during extraction or from degradation byproducts. CAC effectively removes alkanes, alkenes, and aliphatic compounds through hydrophobic interactions, while C_9_H_16_O and C_11_H_22_O_3_ are primarily removed through π–π interactions.^35^ Overall, the application of AC treatment effectively removes organic contaminants from PW, not only improving water quality but also creating a cleaner adsorption environment for subsequent lithium recovery.

In addition to organic removal, pH measurements before and after AC pretreatment and Li/Al-LDH adsorption showed only minor variation across different PW types and solid-to-liquid ratios (Figure S2). The pH generally remained within a neutral to slightly alkaline range, indicating that the AC treatment did not significantly alter the solution chemistry. This suggests that the observed improvements in lithium adsorption are primarily attributed to the removal of interfering species, rather than pH-induced changes in the adsorption environment.

### Mechanistic Insights into LDH Stabilization after AC Pretreatment

The above results indicate that AC exhibits a significant effect in removing organic compounds from PW, which positively influences the subsequent lithium adsorption process by Li/Al-LDH. To further explore the underlying mechanism, this study evaluates the extent of structural degradation of Li/Al-LDH caused by organic compounds in PW, aiming to elucidate their impact on lithium adsorption and provide a theoretical basis for optimizing pretreatment strategies. Figure 4 examines the extent to which organic compounds accumulate within the Li/Al-LDH structure after pretreatment with different types and dosages of AC. This analysis is used to assess the structural damage caused by residual organic contaminants. As shown in Figure 4a, in the treatment of TPW, AC dosages in the range of 0.5–4 g/100 mL effectively reduced the structural damage to Li/Al-LDH caused by organic compounds, thereby playing a protective role. However, when the AC dosage increased to 6 g/100 mL, the structural degradation of Li/Al-LDH exceeded that observed in untreated TPW. This phenomenon can be attributed to the fact that at low AC dosages, AC efficiently adsorbs and removes organic contaminants from PW, mitigating their interference with the layered structure of LDH. However, at an excessive dosage of 6 g/100 mL, the surface functional groups of AC (such as carboxyl and hydroxyl groups) may induce changes in the solution environment, particularly in pH, which adversely affects the stability of Li/Al-LDH during lithium adsorption.^36,37^ This altered chemical environment may lead to the disruption of the LDH interlayer structure upon lithium adsorption.^38,39^ Therefore, using an optimal dosage of AC for TPW treatment can mitigate the structural degradation of Li/Al-LDH caused by the organic compounds during lithium adsorption, thereby potentially enhancing the lithium uptake capacity.

**Figure 4:**
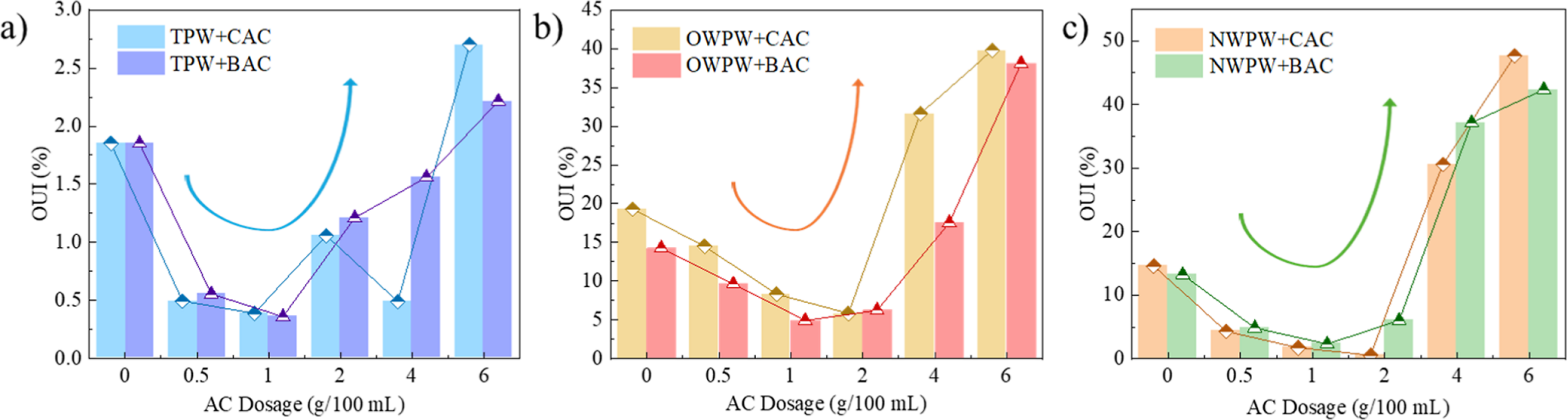
(a) TPW, (b) OWPW, and (c) NWPW: OUI of Li/Al-LDH under different AC treatment types and dosages.

In Figure 4b, a similar trend is observed in the treatment of OWPW, where low AC dosages (0.5–2 g/100 mL) effectively reduce organic-induced structural damage to Li/Al-LDH, whereas higher dosages exert a detrimental effect. This trend aligns with the observations in TPW treatment. However, BAC outperforms CAC in this case. This can be attributed to the prolonged subsurface exposure of OWPW, which results in a higher concentration of humic substances, aromatic hydrocarbons, and other small-molecule organic compounds. The microporous structure of BAC is more effective in adsorbing these persistent organic pollutants, thereby providing better protection for Li/Al-LDH by reducing structural degradation. This conclusion is consistent with the findings presented in Figure S1. Figure 4c illustrates the impact of AC treatment on NWPW. The influence of AC dosage on the structural integrity of Li/Al-LDH follows the same trend as observed in TPW and OWPW, where lower dosages exhibit a protective effect. Additionally, the extent of structural damage or protection provided by BAC and CAC is generally comparable, though CAC shows a slight advantage. In summary, the reason AC treatment of PW facilitates lithium adsorption by Li/Al-LDH lies in its ability to reduce the organic content in PW. By lowering the likelihood of organic compounds coadsorbing into the Li/Al-LDH structure during lithium uptake, AC treatment helps preserve the structural integrity of Li/Al-LDH, thereby enhancing its lithium adsorption efficiency.

To further investigate whether AC pretreatment provides additional benefits beyond organic removal-specifically regarding the structural stability of Li/Al-LDH in PW systems-XRD and FTIR analyses were conducted to evaluate changes in crystal structure and surface functionalities. Figure 5a presents the XRD patterns of Li/Al-LDH after lithium adsorption in the TPW system. Regardless of whether CAC pretreatment was applied, Li/Al-LDH retained its characteristic (003) and (006) crystal planes after lithium adsorption, indicating that its layered structure remained intact.^40^ However, distinct NaCl diffraction peaks (PDF# 05-0628) appeared at around 27°, 32°, 46°, 56°, 65°, and 75°, which can be attributed to the high Na^+^ concentration in TPW (up to 40,000 mg/L).^41^ Competitive adsorption led to Na^+^ incorporation into the Li/Al-LDH interlayer, resulting in the formation of a new NaCl crystalline phase. In this system, CAC pretreatment increased the Figure of Merit (FOM) value of the NaCl diffraction peak from 1.1 to 3.0, indicating a reduced match with the standard NaCl pattern and a lower degree of NaCl crystallization. This suggests that CAC effectively removed a portion of Na^+^ impurities, thereby helping to maintain the standard layered structure of Li/Al-LDH.^42^ In terms of crystalline distance, after lithium adsorption in untreated TPW, the Li/Al-LDH interlayer spacing increased from 7.5349 Å to 7.5977 Å, suggesting that organic compounds in TPW intercalated into the LDH layers, causing expansion.^43^ However, after CAC pretreatment, the interlayer spacing reverted to 7.4849 Å, which closely matches the original Li/Al-LDH structure. This indicates that CAC effectively removed organic contaminants from TPW, minimizing their impact on Li/Al-LDH and preserving its structural integrity. What’s more, Figure 5b illustrates the FTIR spectra of Li/Al-LDH before and after lithium adsorption in TPW with different treatments. The characteristic peaks at 3500 cm^–1^ and 1650 cm^–1^, corresponding to O–H stretching vibrations and H–O–H bending vibrations, respectively, showed minimal changes, indicating that TPW components had a negligible impact on interlayer water.^44,45^ Additionally, the Al–O vibration peak at 1400 cm^–1^ exhibited no significant variations, suggesting that TPW did not substantially alter the functional group composition of Li/Al-LDH.^46^

**Figure 5:**
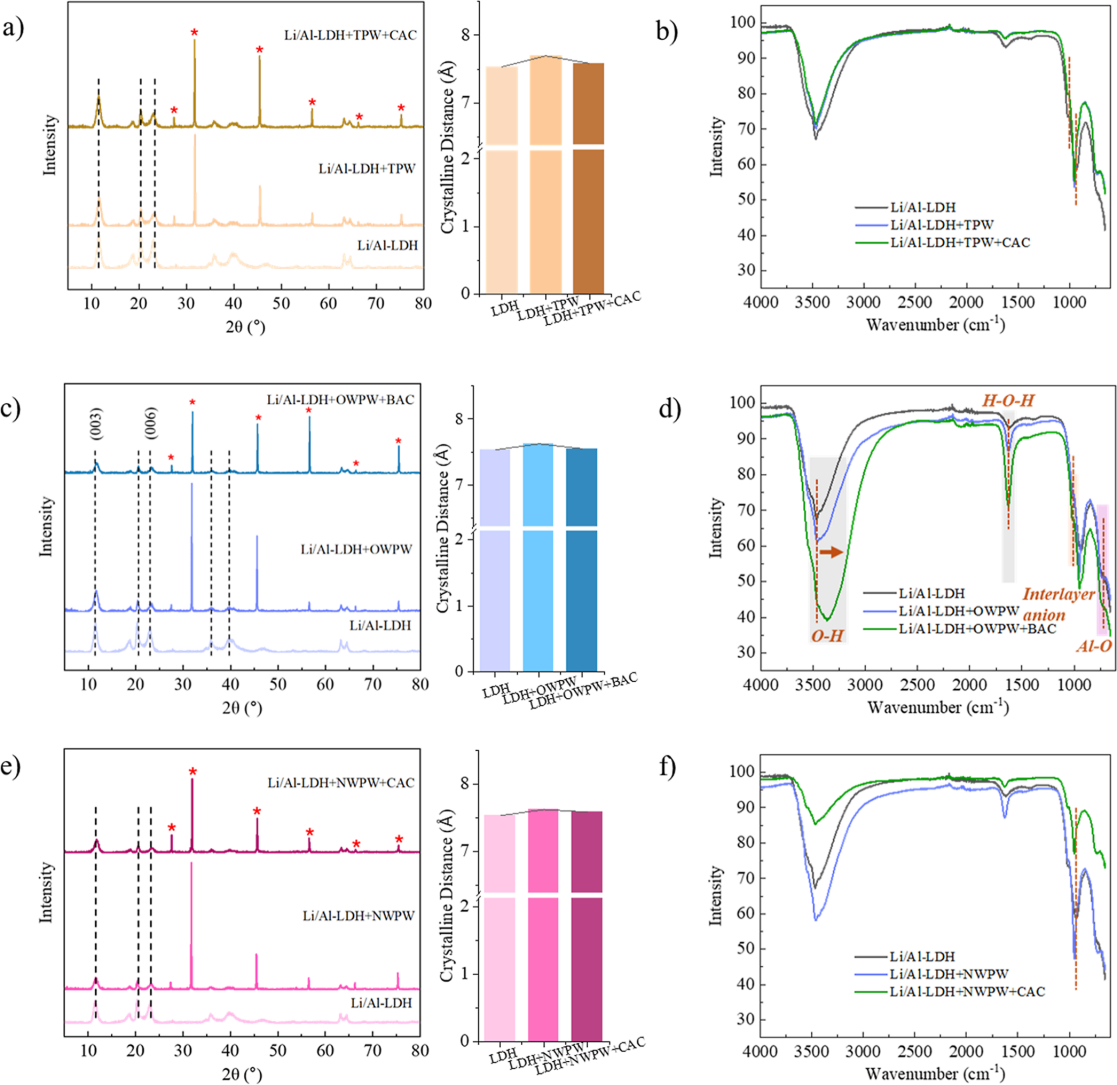
XRD patterns with corresponding interlayer spacing (a,c,e) and FTIR spectra (b,d,f) of Li/Al-LDH before and after adsorption in CAC-/non-CAC-treated TPW, BAC-/non-BAC-treated OWPW, and CAC-/non-CAC-treated NWPW, respectively.

Figure 5c shows the XRD results of Li/Al-LDH in the OWPW system. After lithium adsorption, the layered structure remained intact, with no disruption to the (003) and (006) crystal planes. However, when BAC was used for pretreatment, the NaCl diffraction peak intensity decreased, and the FOM value increased, indicating that the crystal structure tended to revert to the standard Li/Al-LDH phase. This suggests that BAC effectively removed Na^+^ competing ions from OWPW, similar to the effect observed in TPW. Regarding crystalline distance, BAC pretreatment restored the interlayer spacing to a level close to the original structure, further confirming the role of BAC in protecting Li/Al-LDH structural stability in the OWPW system. Figure 5d compares the FTIR spectra of Li/Al-LDH in OWPW with different treatments. In untreated OWPW, the O–H vibration peak at 3250–3500 cm^–1^ exhibited a rightward shift after lithium adsorption, indicating that some OWPW components interacted with interlayer O–H groups. However, after BAC pretreatment, this peak experienced a more pronounced shift, accompanied by a decrease in absorption intensity. This suggests that BAC removed certain organic or inorganic species from the solution, thereby reducing their influence on the Li/Al-LDH interlayer structure. These findings further confirm that BAC pretreatment effectively reduces impurities in OWPW, leading to improved structural stability of Li/Al-LDH.

Finally, Figure 5e presents the results for the NWPW system. Similar to TPW and OWPW, CAC treatment effectively reduced Na^+^ competition and mitigated the impact of organic compounds, helping to maintain the standard Li/Al-LDH structure. However, FTIR analysis in Figure 5f revealed that NWPW had a more pronounced effect on Li/Al-LDH compared to OWPW, as evidenced by greater peak shifts at 3500 cm^–1^ and 1400 cm^–1^. This could be attributed to the higher concentration of dissolved organic matter in NWPW or the presence of inorganic anions such as HCO_3_^–^ and SO_4_^2–^, which could induce more substantial structural changes in LDH.^47^ In contrast, OWPW had been in prolonged contact with subsurface geological formations, which likely led to a more geochemically equilibrated ionic composition. As a result, it exerted a milder impact on the structural integrity of LDH. Meanwhile, TPW underwent dilution during storage, leading to lower impurity concentrations and minimal structural interference. In summary, AC pretreatment effectively reduced the interference of both organic compounds and competing inorganic ions (e.g., Na^+^), thereby improving the structural stability of Li/Al-LDH. This enhanced stability supports more efficient lithium adsorption in PW treatment.

To assess whether AC pretreatment contributes to preserving the morphological integrity of Li/Al-LDH during lithium adsorption, SEM analysis was conducted. As shown in Figure S4, AC pretreatment plays a critical role in protecting the surface morphology of Li/Al-LDH from impurity-induced damage, thereby supporting more efficient lithium adsorption.

To further evaluate the ion removal performance of AC in PW, the concentrations of key metal ions before and after AC treatment were compared, as illustrated in Figure S3. A consistent decrease was observed for K^+^, Mg^2+^, Sr^2+^, Na^+^, and Ca^2+^ across all PW matrices, while Li^+^ concentration remained largely unchanged after AC treatment. As summarized in Figure 6, the removal efficiencies for Ca, Na, K, and Mg were particularly notable-likely due to their relatively high initial concentrations and favorable ionic characteristics (e.g., larger radius and weaker hydration shell), which allow easier adsorption on porous carbon surfaces.^48,49^

**Figure 6:**
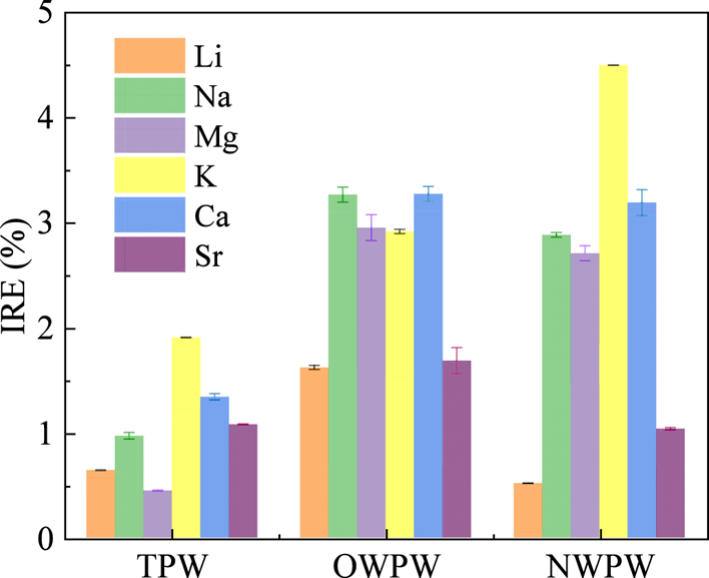
IRE for different metal ions of AC in TPW, OWPW, and NWPW samples.

In contrast, lithium exhibited the lowest removal efficiency (<1%) among all tested ions. Although its low initial concentration might suggest a potential masking effect, this was ruled out by precise ICP–MS measurements (Figure S3a), which showed negligible change in Li^+^ concentration after AC treatment. This confirms that AC has inherently low affinity for Li^+^, likely due to its small ionic radius, strong hydration energy, and weak interaction with AC surface functional groups. These observations collectively indicate that AC does not selectively adsorb Li^+^ and instead prefers other mono- and divalent cations.

Therefore, AC pretreatment removes interfering ions such as Na^+^, K^+^, Ca^2+^, and Mg^2+^ without competing for obvious lithium uptake, thereby facilitating a cleaner solution environment and enabling more selective lithium recovery in the subsequent Li/Al-LDH adsorption process.

### Lithium Recovery Enhancement

To quantify the improvement in lithium adsorption performance resulting from AC treatment, this study systematically investigated the adsorption kinetics and selectivity of Li^+^. As shown in Figure 7a, lithium adsorption in all PW systems followed a distinct trend: an initial rapid adsorption phase within 2–5 min, during which the adsorption capacity increased sharply, followed by a gradual decline in the adsorption rate until the equilibrium was reached at approximately 60 min. This pattern suggests that lithium adsorption was initially limited by the availability of active surface sites, and as these sites became occupied over time, the adsorption rate decreased, eventually stabilizing. Besides, in the TPW system, the lithium adsorption capacity was relatively low, primarily due to the low initial Li^+^ concentration, which resulted in weaker adsorption driving forces. However, after CAC treatment, the lithium adsorption capacity increased from 1.19 mg/g to 1.57 mg/g, indicating that CAC effectively removed organic compounds and other impurities in TPW. This removal process helped mitigate the potential structural degradation of Li/Al-LDH during Li^+^ capture, thereby enhancing adsorption performance. For the OWPW and NWPW systems, AC treatment also improved lithium adsorption, with the most significant increase observed in the NWPW system, where adsorption capacity rose from 2.19 mg/g to 3.12 mg/g, representing a 1.5-fold increase. This improvement was primarily attributed to AC’s exceptional ability to remove impurities from PW systems, which reduced competition and interference from organic compounds and competing ions at the adsorption sites of Li/Al-LDH, thereby increasing lithium adsorption efficiency. Figure 7b illustrates the variation in lithium concentration in PW systems over time. In the initial 2–5 min, the lithium concentration in the solution decreased sharply, which correlated with the rapid uptake of Li^+^ into the layered structure of Li/Al-LDH. As adsorption continued and reached equilibrium after around 60 min, the lithium concentration stabilized. Notably, in CAC-treated NWPW, lithium concentration showed the most significant reduction, decreasing from 85 mg/L to 55 mg/L, further confirming the effectiveness of CAC treatment in enhancing lithium adsorption in PW systems.

**Figure 7:**
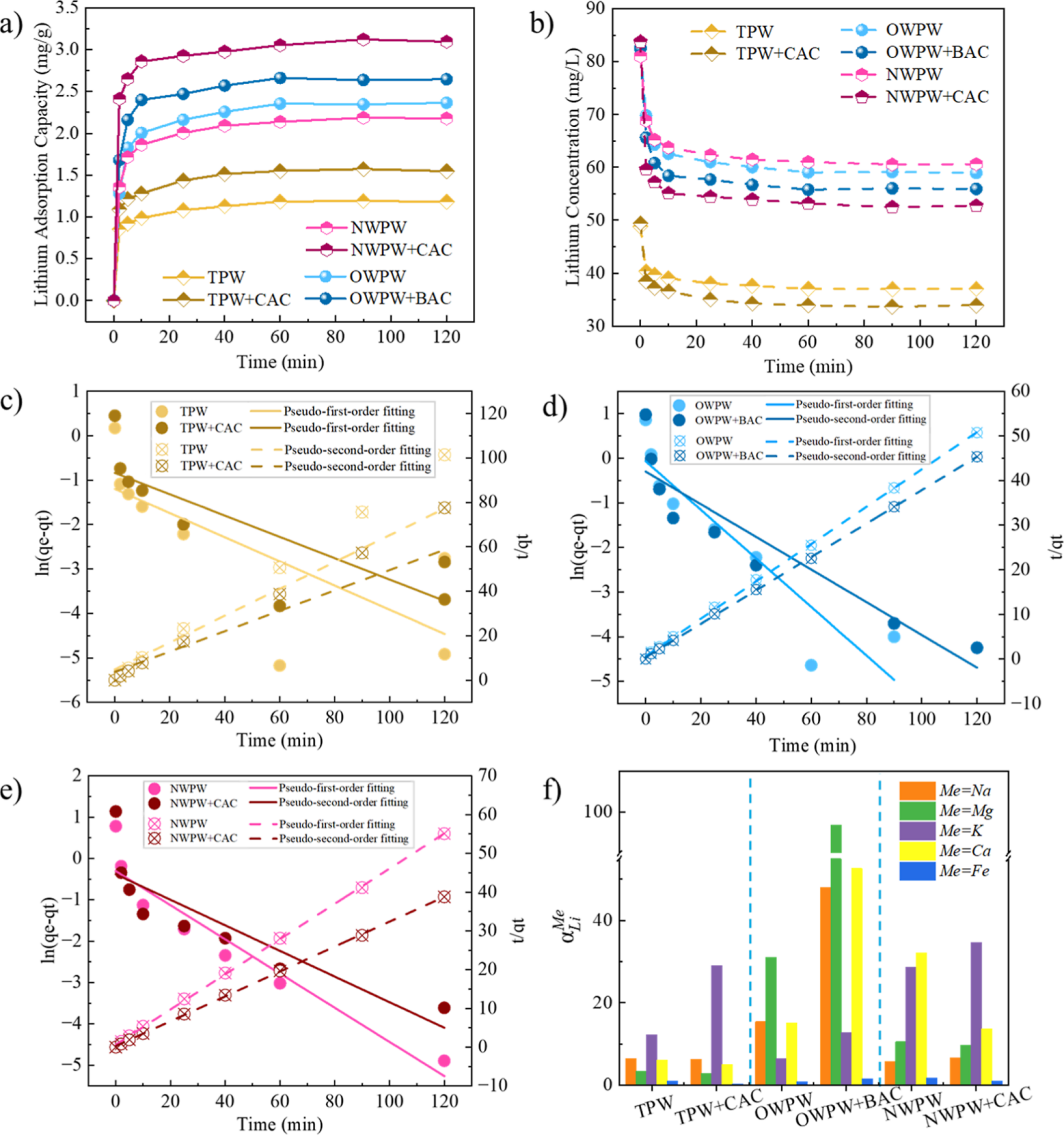
(a) Lithium adsorption kinetics in different PW systems; (b) temporal changes in lithium concentration across systems; (c–e) adsorption kinetics modeling in TPW (c), OWPW (d), and NWPW (e), with and without AC treatment (CAC for TPW and NWPW, BAC for OWPW); (f) Li^+^ selectivity over competing anions in various PW systems.

Subsequently, the adsorption kinetics of Li^+^ in different systems were fitted to kinetic models, with the results presented in Figure 7c–e and Table S3. The fitting results indicated that AC treatment did not significantly alter lithium adsorption kinetics, as all systems conformed to the pseudo-second-order kinetic model. This finding suggests that lithium adsorption was primarily governed by chemisorption rather than physisorption. Of particular interest, in the OWPW and NWPW systems, the pseudo-second-order kinetic model exhibited a *R* ^2^ (high correlation coefficient) of 0.99, further confirming that chemisorption played a dominant role in the adsorption mechanism. Moreover, the *k*_2_ (pseudo-second-order rate constants) in these systems were relatively high, indicating a strong chemical interaction between the adsorbent and Li^+^, involving the intercalation of Li^+^ into the layered structure of Li/Al-LDH.^50^

Importantly, the consistent conformity to the pseudo-second-order model before and after AC pretreatment highlights a key mechanistic insight: the enhancement in lithium recovery is not due to changes in the intrinsic adsorption behavior of Li/Al-LDH, but rather the result of environmental modification—specifically, the removal of interfering organic and ionic species by AC. The relatively high pseudo-second-order rate constants further suggest strong chemical interactions between Li^+^ and the adsorbent surface, likely involving intercalation into the layered structure of Li/Al-LDH. In conclusion, the primary contribution of AC treatment to lithium adsorption lies in impurity removal, rather than altering the structural or chemical properties of Li/Al-LDH.

The selectivity of Li/Al-LDH for Li^+^ adsorption was systematically studied across different systems, revealing significant variations. As shown in Figure 7f, in the TPW system, Li^+^ exhibited a certain degree of selectivity over other metal ions, following the order K^+^ > Na^+^ > Ca^2+^ > Mg^2+^ > Fe^2+^. After CAC treatment, Li^+^ selectivity toward Na^+^ and K^+^ further improved, indicating that CAC effectively removed impurities that interfere with Li^+^ adsorption, thereby enhancing the selectivity of the adsorbent for monovalent metal ions. In the OWPW system, Li^+^ showed high selectivity for Mg^2+^, Ca^2+^, Na^+^, with *a*_Li_^Mg^exceeding 80 and *a*_Li_^Ca^ around 60. This phenomenon is directly related to ion radius, as the vacancies size of Li/Al-LDH layer is primarily suited for Li^+^, preventing larger divalent ions such as Mg^2+^ and Ca^2+^ from entering the structure, thus resulting in higher selectivity for Li^+^.^39^ After BAC treatment, Li^+^ selectivity improved for all metal ions, regardless of their valency. This enhancement is attributed to BAC’s dual function: not only does it remove organic matter, preserving the structural integrity of Li/Al-LDH and preventing adsorption site degradation, but it also adsorbs certain competing ions, such as Na^+^, reducing their interference with Li^+^ adsorption and further improving selectivity. In addition, in the NWPW system, Li^+^ selectivity was relatively balanced, with ion rejection falling between that observed in TPW and OWPW. However, after CAC treatment, Li^+^ selectivity toward K^+^ and Ca^2+^ increased significantly, particularly *a*_Li_^K^ and *a*_Li_^Ca^, demonstrating that CAC effectively removes coexisting ions that interfere with Li^+^ adsorption, thereby reducing competition from K^+^ and Ca^2+^ and enhancing Li^+^ selectivity. These findings highlight the system-dependent effect of AC treatment on Li^+^ selectivity. Specifically, CAC treatment in the NWPW system showed the most significant improvement, particularly in reducing interference from K^+^ and Ca^2+^, while in the TPW system, CAC mainly enhanced selectivity for Na^+^ and K^+^, though the overall improvement was relatively limited. In contrast, BAC treatment in the OWPW system improved Li^+^ selectivity but was less effective at removing competing metal ions compared to CAC treatment in the NWPW system. Furthermore, multicycle experiments in Figure S5 confirmed that Li/Al-LDH retained a stable lithium adsorption capacity over at least six regeneration cycles, especially when preceded by AC pretreatment, demonstrating its promising recyclability for long-term use. Overall, AC treatment optimizes the adsorption environment of Li/Al-LDH by removing impurities and reducing competition from coexisting ions, thereby enhancing Li^+^ selectivity, with the most pronounced effect observed in the NWPW system.

### Mechanistic Insights

Figure 8 illustrates the role of AC in different PW systems and its synergistic effect on the subsequent Li/Al-LDH adsorption of Li^+^. Mechanistically, variations in the composition and concentration of organic matter across PW sources necessitate effective pretreatment to improve adsorption performance. AC, owing to its highly porous and hydrophobic structure, effectively removes organic impurities from the aqueous phase. This not only reduces interference during Li^+^ adsorption but also significantly improves overall water quality by lowering the organic load.

**Figure 8:**
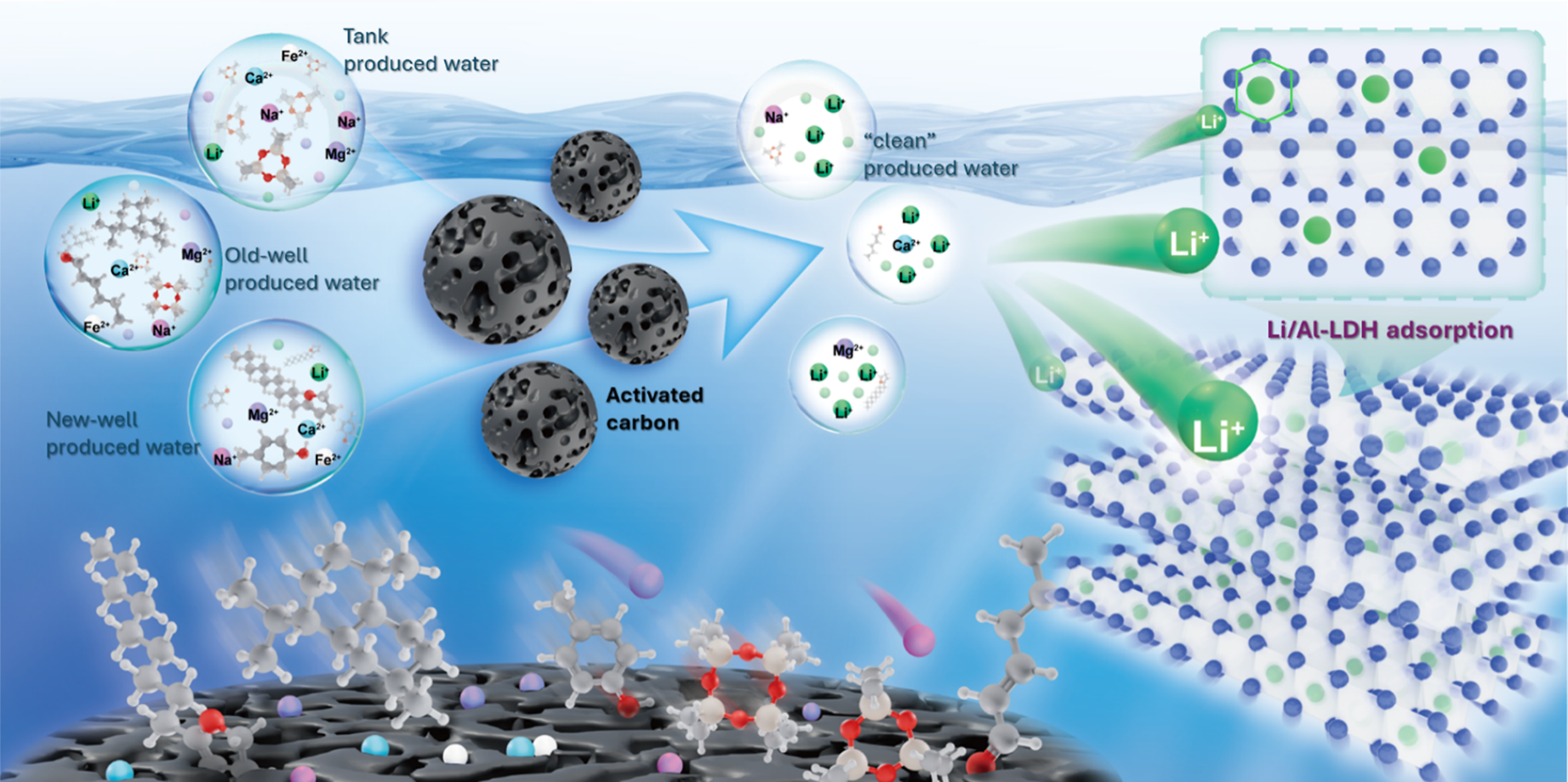
Diagram of enhanced lithium extraction from three PW sources using activated carbon.

The removal of organic contaminants is governed by the balance between van der Waals (vdW) attraction and electric double layer (EDL) repulsion, as described by DLVO theory.^51,52^ AC’s hierarchical porosity and hydrophobic surface amplify vdW forces, enabling efficient adsorption of nonpolar compounds such as aliphatic hydrocarbons.^53^ However, when AC and organics share similar surface charges (e.g., negative charges at pH > pH_p*zc*_), EDL repulsion may hinder the uptake of charged species like humic acids. This vdW–EDL interplay is further modulated by three key factors: (1) surface charge compatibility, enhanced under acidic conditions or in the presence of divalent cations (e.g., Ca^2+^); (2) hydrophobic interactions that favor adsorption of nonpolar species; and (3) ionic strength, where high salinity compresses the EDL, enhancing vdW dominance and overall adsorption efficiency.^54^

The resulting cleaner solution environment minimizes competitive adsorption, reduces the risk of surface fouling, and protects the structural integrity of the subsequent adsorbent material. Following AC pretreatment, purified PW enters the Li/Al-LDH system, where Li^+^ is selectively adsorbed into layer vacancies with favorable ionic size compatibility. The presence of AC thus enhances lithium adsorption through two synergistic pathways: (1) reducing nonspecific interactions by removing interfering species, and (2) mitigating structural degradation, thereby improving long-term adsorption stability.

## Conclusion

This study presents an integrated and sustainable strategy that combines activated carbon pretreatment with Li/Al-LDH adsorption for produced water treatment, demonstrating dual benefits of water quality improvement and lithium recovery enhancement. AC pretreatment effectively removes organic contaminants and competing cations, thereby reducing fouling and creating a more favorable environment for Li^+^ uptake. In addition, it preserves the crystalline structure and surface morphology of Li/Al-LDH, enhancing its stability and extending its functional lifespan. Lithium recovery was significantly improved after AC treatment, while the intrinsic kinetic behavior remained unchanged, indicating that AC functions as a sustainability-enabling preconditioning step without chemically altering the adsorbent.

From a sustainability perspective, the proposed approach is aligned with green chemistry and circular economy principles. The ACs used—derived from agricultural or industrial byproducts such as coconut shells and coal residues—support the reuse of waste materials and minimize reliance on virgin feedstocks. The low material dosage, high adsorption capacity, and regeneration potential of both AC and Li/Al-LDH under neutral pH conditions reduce operational cost, energy input, and secondary pollution, contributing to a low-impact process design. Moreover, spent ACs can be regenerated through established thermal or chemical methods, allowing for multiple reuse cycles and further reducing waste generation and material input. The pretreatment-adsorption integration addresses two environmental challenges simultaneously: the remediation of high-salinity, organic-rich industrial wastewater and the recovery of a critical mineral (Li^+^), supporting a more circular and resilient lithium supply chain. Additional discussion on the process scalability and envisioned implementation pathway is provided in the Supporting Information (Figure S6).

Overall, this work advances a scalable, resource-efficient, and environmentally benign solution for critical mineral recovery from complex wastewaters. In future work, the regeneration performance and long-term recyclability of AC will be further evaluated to strengthen the full-cycle sustainability of the process. By enhancing material performance while reducing chemical inputs and waste generation, it offers a technically robust and ecologically responsible pathway that supports long-term sustainability goals in clean energy and water management sectors.

## Supplementary Material



## Notes

Ion concentrations in the different produced water samples (Table S1); organic composition and pretreatment purpose for the different produced water types (Table S2); 2. adsorption kinetic models and selectivity factors (Equation S1–S4); N_2_ adsorption–desorption isotherms of both Acs, Surface area, pore volume, and pore size distribution of ACs (Figure S1); pH variation in (a) OWPW, (b) NWPW, and (c) TPW following different AC treatments and Li/Al-LDH adsorption (Figure S2); ion concentration comparison before and after AC treatment in different produced water samples: (a) Li and K, (b) Mg and Sr, (c) Na and Ca (Figure S3); morphologies of Li/Al-LDH before and after lithium adsorption in raw and AC-treated TPW (a), OWPW (b), and NWPW (c), respectively (Figure S4); kinetic fitting parameters of lithium adsorption process in different produced water systems (Table S3); lithium adsorption/desorption cyclic performance (Figure S5); proposed scalable process flow for lithium recovery from produced water, integrating activated carbon (AC) pretreatment and Li/Al-LDH adsorption (Figure S6) (PDF)
