# Supplementary material for: Impurity Shielding in Li/Al-LDH-Based Lithium Recovery from Produced Water via Activated Carbon Pretreatment
Source: ACS Sustain Chem Eng. 2025 Aug 25;13(35):14527–39. doi: 10.1021/acssuschemeng.5c05254 (PMC12421675; doi:10.1021/acssuschemeng.5c05254)
Supplement: Supplementary file 1 [file sc5c05254_si_001.pdf]

Supporting Information

Impurity Shielding in Li/Al-LDH-based Lithium Recovery from Produced Water via Activated Carbon Pretreatment

Yanan Pan [a], Wencai Zhang [a,*]

[a] *Department of Mining and Minerals Engineering, Virginia Polytechnic Institute and State University, Blacksburg, Virginia 24061, USA*

Corresponding author:

Dr. Wencai Zhang (E-mail: wencaizhang@vt.edu)

Number of pages: 12

Number of figures: 6

Number of tables: 3

1. Properties of produced water samples

Table S1 Ion concentrations in the different produced water samples.

Ion Concentration	OWPW	NWPW	TPW
Mn (ppm)	17.68	12.77	5.03
Fe (ppm)	142.99	149.58	70.12
Na (ppm)	41831.37	41415.68	25501.80
Al (ppm)	46.25	46.83	45.69
B (ppm)	109.55	113.69	117.62
Ca (ppm)	23499.13	20985.46	10993.52
K (ppm)	394.03	393.85	528.21
Mg (ppm)	2154.05	1981.93	1083.10
S (ppm)	439.63	333.86	263.12
Si (ppm)	92.69	139.70	137.08
Sr (ppm)	3080.66	2850.94	1968.15

Table S2. Organic composition and pretreatment purpose for the different produced water types.

Produced Water Type	Main Organic Constituents	Likely Origin or Cause	Purpose of Pretreatment
TPW (from a 50,000-gallon storage tank)	Soluble organics; moderate DOM content	Residual treatment-stage compounds and partial degradation products from centralized processing and storage	Remove remaining soluble organics to reduce background interference and enhance lithium adsorption efficiency
OWPW (13-14 years of operation)	Humic substances, aromatic hydrocarbons, persistent organic compounds	Prolonged subsurface exposure leading to accumulation of complex natural organic matter and reservoir-derived aromatics	Remove humic/aromatic organics that interfere with Li/Al-LDH uptake, improving lithium adsorption
NWPW (1-2 years of operation)	Short-chain hydrocarbons, crude oil residues, surfactant-like compounds; low DOM	Limited formation interaction; presence of production-phase oil residues and chemical additives	Remove small hydrocarbon and surface-active compounds to optimize aqueous phase for lithium adsorption

2. Adsorption kinetic models and selectivity factors

$$ln(q_e - q_t) = ln q_e - k_1 \cdot t \tag{S1}$$

$$\frac{t}{q_t} = \frac{1}{k_2 \cdot q_e^2} + \frac{1}{q_e} \tag{S2}$$

where q_e (mg/g) means the equilibrium lithium adsorption capacity and q_t (mg/g) means the capacity at time t. k_1 (1/min) and k_2 (g/(mg·min)) are the rate constant of these two models, respectively.

$$K_{Me} = \frac{(C_0 - C_e) \cdot v}{C_e \cdot m} \tag{S3}$$

$$a_{Li}^{Me} = \frac{K_{Li}}{K_{Me}} \tag{S4}$$

where C_0 (mg/L) and C_e (mg/L) refer to then initial ion concentration and the equilibrium concentration, m (g) is the added adsorbents mass, v (L) is the solution volume. The subscript M_e of K_{Me} and a_{Li}^{Me} refer to the metal ions in the solution.

3. Activated carbon characterizations

Figure S1(a) presents the N_2 adsorption-desorption isotherms, where both materials exhibit type IV hysteresis loops, characteristic of capillary condensation in mesopores, with adsorption saturation occurring at high relative pressures (P/P_0)[1]. The presence of enhanced microporosity is evident from the pronounced knee region in the low-pressure range of the isotherms, followed by a steeper adsorption slope in the intermediate pressure range. Figure S1(b) comparatively illustrates the textural parameters of both ACs. The CAC demonstrates superior specific surface area (exceeding 200 m^2/g) and larger pore volume (approximately 0.11 cm^3/g) compared to BAC, suggesting a more developed porous structure that can provide greater adsorption capacity per unit mass. CAC exhibits a hierarchical pore structure with a significant presence of mesopores and macropores, while pore size distribution analysis reveals that both materials have average pore widths around 2.5 nm, positioned at the transition between micropores and mesopores. This bimodal distribution confirms the coexistence of microporous and mesoporous characteristics in both ACs. The marginally smaller average pore diameter of BAC implies a higher proportion of micropores.

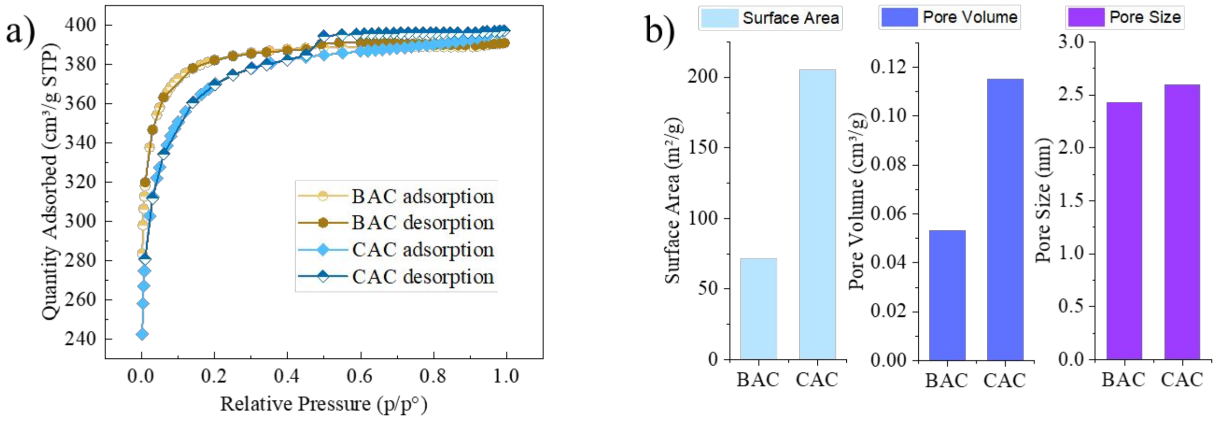

Figure S1. (a) N_2 adsorption-desorption isotherms of both ACs; (b) Surface area, pore volume, and pore size distribution of ACs.

4. pH variation before and after AC treatment

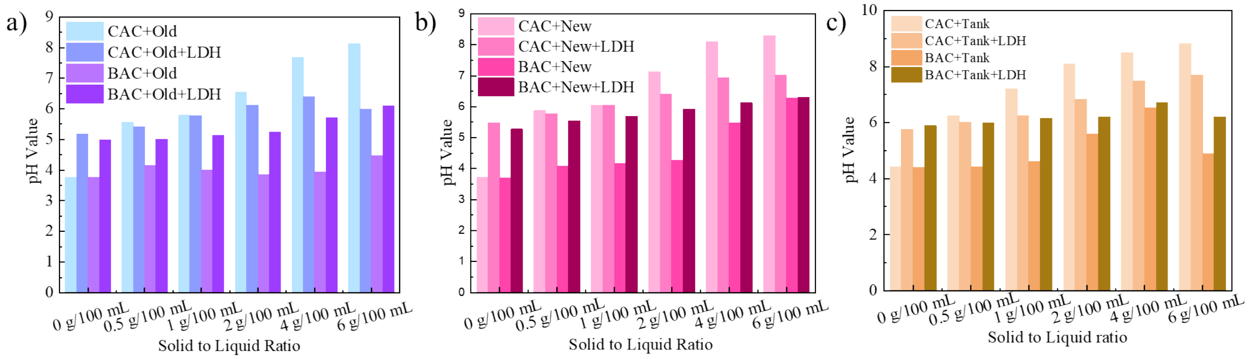

Figure S2. pH variation in (a) OWPW, (b) NWPW, and (c) TPW following different AC treatments and Li/Al-LDH adsorption.

5. Metal ions concentration comparison before and after AC treatment

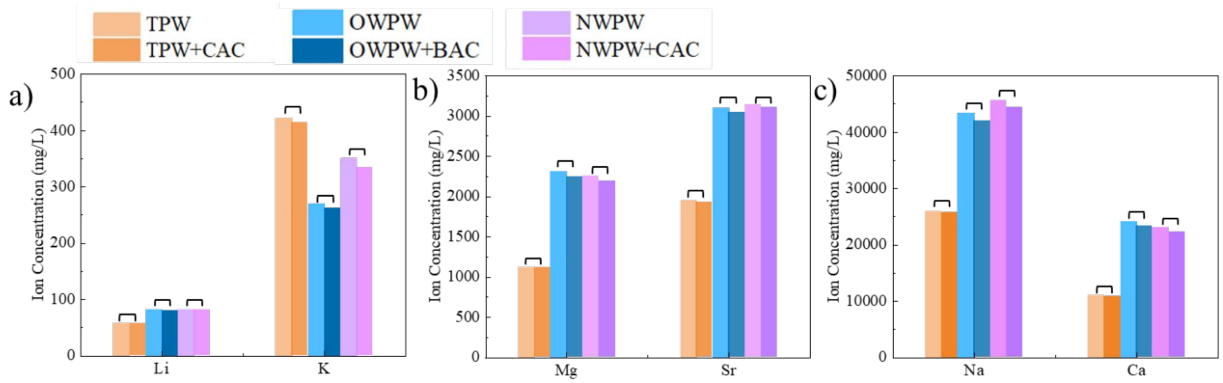

Figure S3. Ion concentration comparison before and after AC treatment in different produced water samples: (a) Li and K, (b) Mg and Sr, (c) Na and Ca.

6. Morphology comparison of LDH adsorbent before and after AC treatment

Figure S4(a) illustrates the morphological changes of Li/Al-LDH before and after lithium adsorption in the TPW system. Before adsorption, Li/Al-LDH exhibits a typical layered nanosheet structure with a uniform surface and clearly defined layers[2]. The magnified image reveals tightly interconnected nanosheets with an intact layered structure. However, after lithium adsorption in untreated TPW, the layered structure undergoes significant degradation, with the appearance of irregular particles and deposits on the surface. The magnified image shows a more dispersed layer distribution and increased surface roughness, indicating that impurities in PW severely impact the morphology of Li/Al-LDH. In contrast, after lithium adsorption in CAC-pretreated TPW, the layered structure partially recovers, with reduced structural damage, clearer layer alignment, and improved surface smoothness. This suggests that CAC pretreatment, by removing organic contaminants and competing ionic species such as Na^+, mitigates the structural disruption caused by PW and helps preserve the morphological integrity of Li/Al-LDH.

Similarly, Figure S4(b) presents the morphological changes in the OWPW system. After lithium adsorption in untreated OWPW, the Li/Al-LDH layered structure experienced severe degradation. The magnified image reveals uneven layer distribution, with some layers appearing partially peeled or dissolved, which may be attributed to the high concentration of complex organic compounds in OWPW[3]. However, after BAC pretreatment, the Li/Al-LDH layered structure shows partial recovery. Although some deposits remain, the integrity of the layers improves compared to the untreated OWPW sample. This indicates that BAC treatment helps reduce impurity deposition and protects the LDH layers from further degradation.

Figure S4(c) depicts the morphological changes in the NWPW system. After lithium adsorption in untreated NWPW, the interlayer spacing of Li/Al-LDH increases significantly, likely due to partial dissolution or destruction of the layers. Additionally, a higher amount of surface deposits is observed, suggesting that NWPW may contain substances that promote aggregation or scaling, leading to impurity accumulation on Li/Al-LDH. Following CAC pretreatment, the layered structure of Li/Al-LDH partially recovers compared to the untreated NWPW sample, with fewer surface deposits and more clearly defined layered nanosheets.

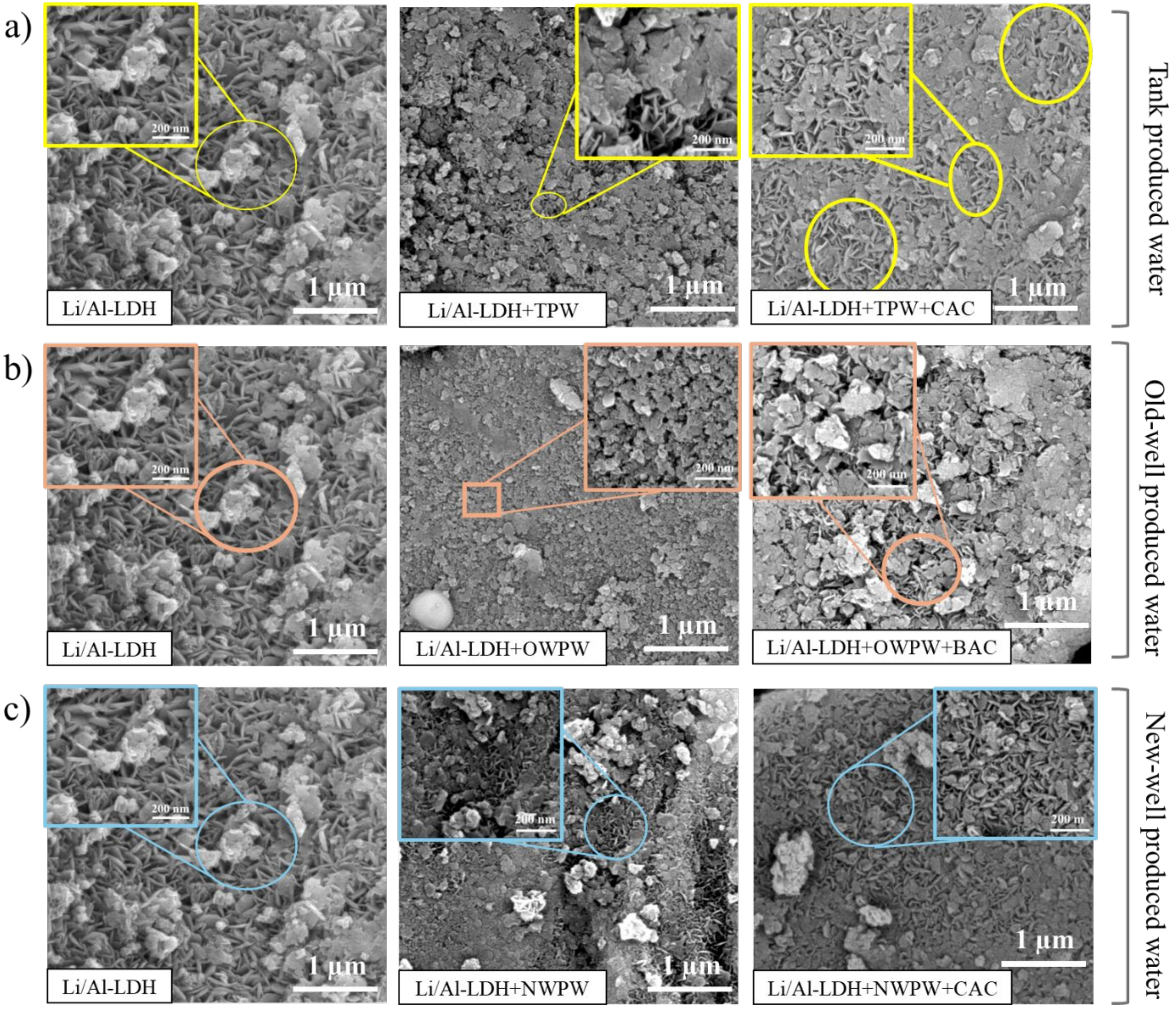

Figure S4. (a-c) Morphologies of Li/Al-LDH before and after lithium adsorption in raw and AC-treated TPW (a), OWPW (b), and NWPW (c), respectively.

7. Kinetic fitting parameters and cyclic performance

Table S3. Kinetic fitting parameters of lithium adsorption process in different produced water systems.

	Q (mg/g)	pseudo-first-order model			pseudo-second-order model		
		q_{e1} (mg/g)	k_1 (1/min)	R_1^2	q_{e2} (mg/g)	k_2 (g/(mg·min))	R_2^2
TPW	1.19	0.7321	0.0272	0.4907	0.60356	0.0754	0.6928
TPW+CAC	1.57	0.8612	0.0240	0.6177	0.45817	0.05686	0.6853
OWPW	2.36	0.4131	0.0544	0.8244	0.41845	0.2681	0.9996
OWPW+BAC	2.66	0.4312	0.0366	0.8379	0.37456	0.3599	0.9998
NWPW	2.19	0.4331	0.0413	0.8979	0.45389	0.4539	0.9997
NWPW+CAC	3.12	0.4212	0.0309	0.7213	0.32064	0.3560	0.9997

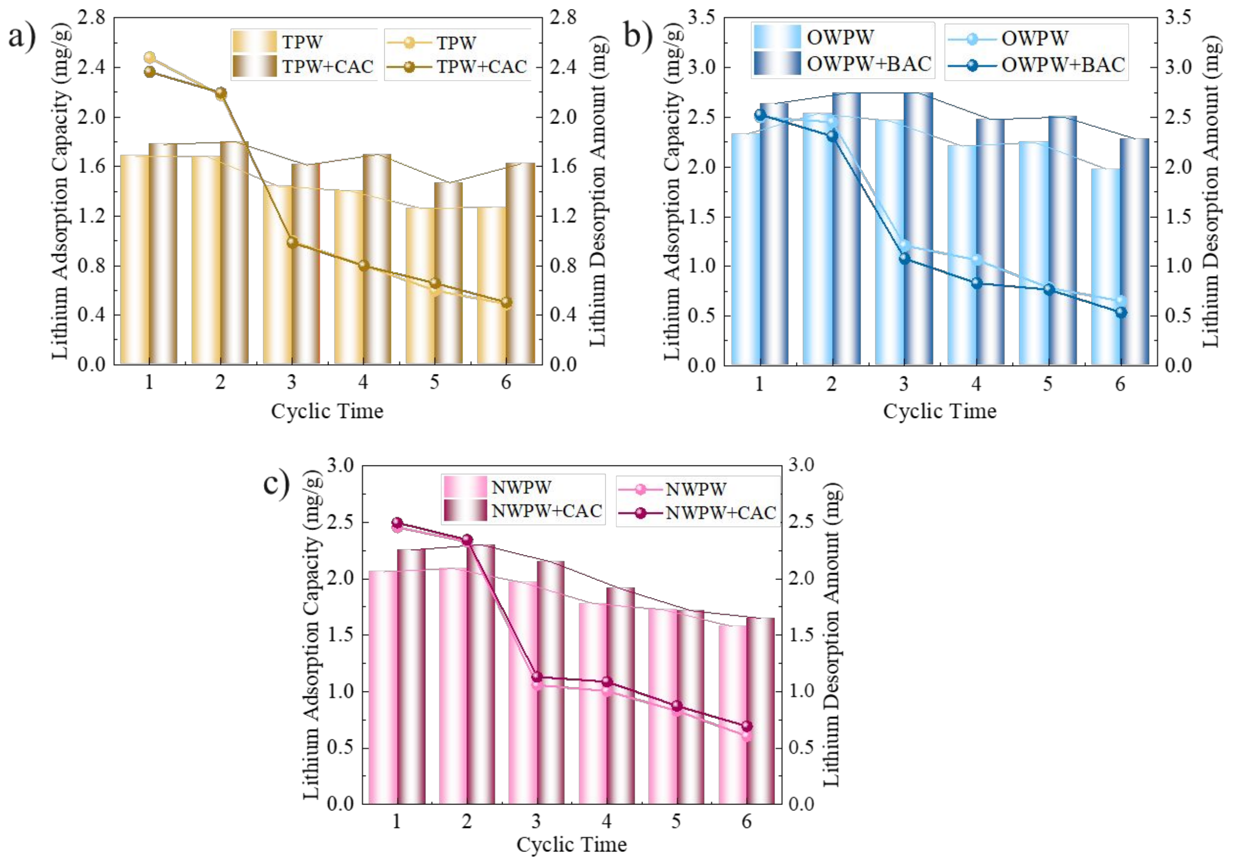

Figure S5. Lithium adsorption/desorption cyclic performance in (a) TPW and TPW+CAC, (b) OWPW and OWPW + BAC, and (c) NWPW and NWPW + CAC.

8. Process Scalability and Sustainable Implementation Considerations

The proposed AC + Li/Al-LDH process exhibits strong potential for scalable and sustainable deployment. Both materials can be synthesized from low-cost, widely available feedstocks—AC from agricultural or industrial waste (e.g., coconut shells, coal residues), and Li/Al-LDH via mild co-precipitation methods. The process lends itself to modular implementation: AC pretreatment can be carried out in packed-bed columns or stirred tanks, followed by Li/Al-LDH adsorption in fixed-bed systems. Both materials are regenerable under environmentally benign conditions—AC through thermal or chemical methods, and Li/Al-LDH via mild desorption without generating secondary pollution. These features allow for closed-loop material use and integration into existing water treatment frameworks. A conceptual process flow is provided in Figure S6. Future work will focus on pilot-scale testing, regeneration performance, and life-cycle/techno-economic analysis to optimize the system for industrial application.

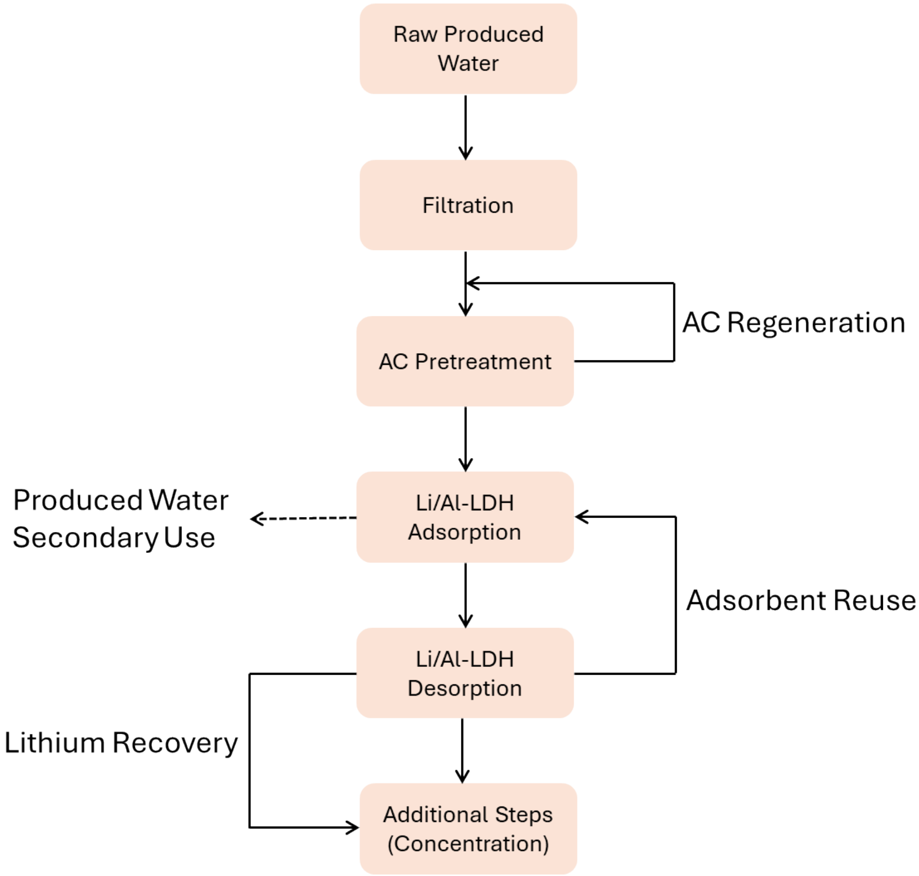

Figure S6. Proposed scalable process flow for lithium recovery from produced water, integrating AC pretreatment and Li/Al-LDH adsorption.

Reference

(1) Lua, A. C. A comparative study of the pore characteristics and phenol adsorption performance of activated carbons prepared from oil-palm shell wastes by steam and combined steam-chemical activation. *Green Chemical Engineering* **2024**, *5* (1), 85-96, DOI: 10.1016/j.gce.2022.11.004.

(2) Yu, J.; Tang, M.; Cui, P.; Deng, J.; Mi, H.; Zhang, L.; Chen, L.; Wu, P.; Chao, Y.; Zhu, W.; et al. Computational fluid dynamics and machine learning assisted Al-LDH adsorbent reactor design for lithium recovery from salt lakes. *Desalination* **2025**, *600*, 118396, DOI: 10.1016/j.desal.2024.118396.

(3) Pan, Y.; Ji, B.; Zhang, W.; Knott, K.; Xia, Y.; Li, Q.; Rena, B. Topography and structural regulation-induced enhanced recovery of lithium from shale gas produced water via polyethylene glycol functionalized layered double hydroxide. *Journal of Industrial and Engineering Chemistry* **2024**, DOI: 10.1016/j.jiec.2024.10.032.
